# Synthesis of n-type Mg_2_Si/CNT Thermoelectric Nanofibers

**DOI:** 10.1186/s11671-017-2120-y

**Published:** 2017-05-10

**Authors:** Keiko Kikuchi, Kodai Yamamoto, Naoyuki Nomura, Akira Kawasaki

**Affiliations:** 1 Department of Materials Processing, Graduate School of Engineering, Tohoku University, Sendai, 980-8579 Japan; 2 Postal address: 6-6-02, Aramaki Aza Aoba, Aoba-ku, Sendai, Miyagi 980-8579 Japan

**Keywords:** Carbon nanotubes, Magnesium silicide, Thermoelectric nanofibers, Flexible thermoelectric material

## Abstract

Magnesium silicide (Mg_2_Si)/carbon nanotube (CNT) thermoelectric nanofibers for use as a flexible thermoelectric material were successfully synthesized through the combined processes of the sol-gel method, magnesiothermic reduction, and liquid-solid phase reaction. In the resulting product, each CNT was coated with Mg_2_Si which was an approximately 60-nm-thick single crystal. The synthesized Mg_2_Si-coated CNTs exhibited n-type thermoelectric behavior confirming that n-type thermoelectric composite nanofibers were successfully obtained.

## Background

Thermoelectric (TE) devices have attracted much attention as energy harvesting systems because they are able to convert thermal energy directly to electrical energy [1, 2]. In recent decades, most TE research has focused on inorganic materials such as semiconductors or conducting oxides [1–3]. Recently, in addition to rigid inorganic materials, flexible organic materials have been receiving much attention as TE materials that may provide mechanical flexibility and low manufacturing cost [4–6]. Conducting polymers such as poly(3,4-ethylenedioxythiophene):poly(styrenesulfonate) (PEDOT:PSS) exhibit attractive thermoelectric properties around room temperature [7–10]. However, they are unstable at temperatures over 500 K, so their operating temperature is somewhat restricted [9, 10]. For flexible TE materials that can be used over 500 K, carbon nanotubes (CNTs) are a promising candidate because of their large Seebeck coefficient as exhibited in CNT films in addition to their superior electrical properties, chemical stability, good mechanical strength with excellent flexibility, and thermo-oxidative stability up to 873 K [11–13]. However, most studies on TE materials consisting of CNTs have reported on only p-type samples and there are only a few studies on that of n-type samples, because n-doped CNTs are easily oxidized in air [14, 15]. It is well known that p-n-connected TE devices are capable of efficiently generating electric power. Consequently, n-type flexible TE materials that can be used at temperatures above 500 K have been in high demanded. Inorganic materials exhibiting a stable and high TE effect even at high temperature [16] are mainly semiconductors and are inherently rigid and brittle. Thus, it is difficult to fabricate a flexible film directly from inorganic TE materials. If CNTs with good mechanical strength and high electrical conductivity are individually coated with a stable, but rigid and brittle, inorganic n-type TE material, it is expected that the obtained composite nanofibers would become high-strength, thermally stable, n-type TE nanofibers, and a flexible TE film could be fabricated from those composite TE nanofibers.

In this study, we synthesize a new composite nanofiber where each CNT is partially coated with an inorganic TE material. We further propose that an n-type TE film made from these composite nanofibers could serve as a flexible TE material that can be used at relatively high temperature, i.e., above 500 K. Magnesium silicide (Mg_2_Si) was selected as the TE material for coating because not only are Mg and Si both non-toxic and abundant elements but Mg_2_Si has superior thermo-oxidative stability up to around 720 K [17, 18].The Mg_2_Si coating was successfully synthesized on individual CNTs via a three-step process consisting of (1) formation of a silica coating on the CNTs by the sol-gel method [19–21], (2) magnesiothermic reduction [21–24] of the silica coating to a silicon (Si) coating, and (3) synthesis of a Mg_2_Si coating by a liquid-solid phase reaction [25] of the silicon layer with Mg powder. Subsequently, measurement of TE properties revealed that the synthesized Mg_2_Si-coated CNT nanofibers exhibited n-type thermoelectric behavior.

## Methods

Multiwalled carbon nanotubes with diameters of 20–70 nm, which were synthesized by catalytic chemical vapor deposition (CVD) and subsequently annealed above 2473 K (Hodogaya Chemical Co., Ltd.) [13], were suspended in a 3:1 mixture of concentrated H_2_SO_4_ and HNO_3_ (v/v) under ultrasonication in a water bath at 323 K for 24 h [26]. The resultant suspension was diluted with deionized water and filtered to collect acid-treated CNTs on a filter paper. After washing with deionized water and ethanol, they were dried at 323 K in air. Then, 0.4 mg of the acid-treated CNTs was dispersed in 160 ml of ethanol by ultrasonication for 1 h. Subsequently 20 ml of deionized water, 2 ml of tetraethylorthosilicate (TEOS), and 35 ml of 25% NH_4_OH were added to the suspension. The acid-treated CNTs were chemically coated with amorphous silica during 1 h of stirring. The obtained silica-coated CNTs were washed with ethanol, filtered, and dried overnight at room temperature. The silica-coated CNTs were mixed with magnesium powder with an average particle diameter of 180 μm (Kojundo Chemical Laboratory Co., Ltd.) at a molar ratio of silica to magnesium of 2:1. The amount of silica on the silica-coated CNTs was calculated from the geometry based on the thickness of the silica layer and assuming an average CNT diameter of 40 nm. The mixed powders were sealed in a steel tube with N_2_ gas. Then, the tube was heated to 1023 K in a tube furnace and held for 5 h in flowing N_2_ gas. The silica coating on the CNTs was reduced to a Si coating during this heat-treatment; however, treatment with 20 wt% HCl was necessary to remove by-products from the reacted mixture. Finally, the Si-coated CNTs were mixed with magnesium powder at a molar ratio of Si to magnesium of 3:1. The amount of Si in the Si-coated CNTs was assumed to correspond with the amount of Si resulting from complete reduction of the original silica coating. The mixed powders were sealed in a steel tube with Ar gas. The tube was heated to 973 K at a heating rate of 5 K/min in a tube furnace, held for 2 h in flowing Ar gas, and then cooled down to room temperature at a cooling rate of 1 K/min. This process allowed the Si coating to react with Mg resulting in Mg_2_Si-coated CNTs.

The obtained products were evaluated by X-ray diffraction (XRD) with Cu K_α_ radiation (RINT, Rigaku Corporation) and field-emission transmission electron microscopy (FE-TEM) with energy dispersive X-ray spectroscopy (EDX) (JEM-2100F, JEOL Ltd.). In order to obtain a bar-shaped specimen large enough to measure thermoelectric properties, acid-treated CNTs and Mg_2_Si-coated CNTs were pressed in a steel die, respectively, and then a mixture of epoxy resin and curing agent (CY221 and HY956, Nagase ChemteX Corporation; 5:1 by weight) was infiltrated into the pressed compacts followed by curing at room temperature for 24 h. The obtained compacts were cut into a rectangle (2 × 2 × 8 mm), and the Seebeck coefficient and electrical resistivity were measured using a ZEM-3 instrument (ADVANCE RIKO, Inc.) at room temperature. The maximum temperature difference applied to the sample during the Seebeck coefficient measurement was 30 K.

## Results and Discussion

**Fig. 1:**
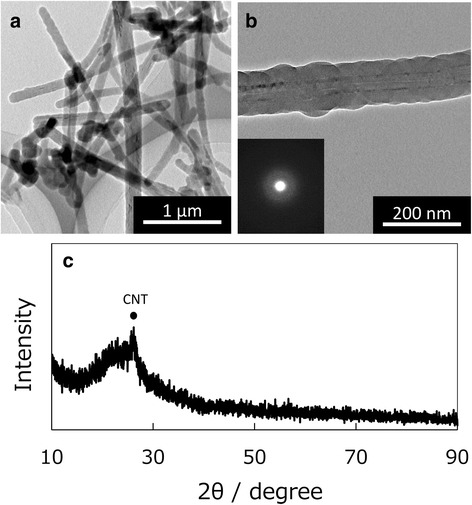
TEM images (**a**, **b**) and XRD profile (**c**) of silica-coated CNTs

Figure 1 shows TEM images and an XRD profile of silica-coated CNTs. A uniform, continuous, 50-nm-thick coating was synthesized on individual CNTs (Fig. 1a and b). The EDX analysis revealed that this coating consisted of Si and O. The very diffuse ring visible in the selected-area electron diffraction (SAED) pattern of the coating (inset of Fig. 1b) and the broad peak in the XRD pattern (Fig. 1c) suggest that the synthesized layer is amorphous silica.

**Fig. 2:**
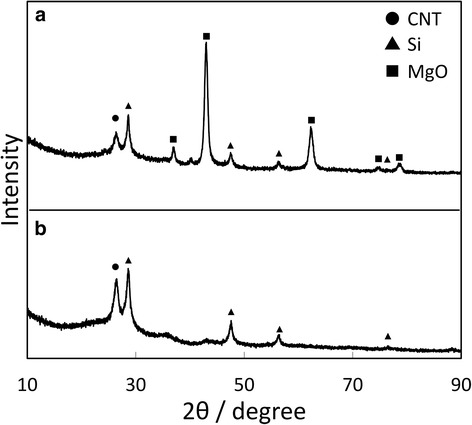
XRD profile of the composite nanofibers after magnesiothermic reduction (**a**) and subsequent HCl treatment (**b**)

Figure 2a shows an XRD profile of the nanofibers after magnesiothermic reduction of the silica-coated CNTs. Diffraction peaks from Si and magnesium oxide (MgO) as well as a peak from the CNTs were observed, which indicates the reduction occurred according to the following reaction [22]:

1 $$ 2\mathrm{Mg}\left(\mathrm{g}\right)+{\mathrm{SiO}}_2\left(\mathrm{s}\right)\to\ 2\mathrm{M}\mathrm{g}\mathrm{O}\left(\mathrm{s}\right)+\mathrm{S}\mathrm{i}\left(\mathrm{s}\right) $$

Figure 2b shows an XRD profile of the reduced nanofibers after HCl treatment. The major peaks originate from CNT and Si. This result indicates that MgO is a by-product of the magnesiothermic reduction of silica and was removed by HCl treatment. From these XRD profiles, it is clear that silica was reduced to Si, and Si-coated CNTs were successfully obtained.

**Fig. 3:**
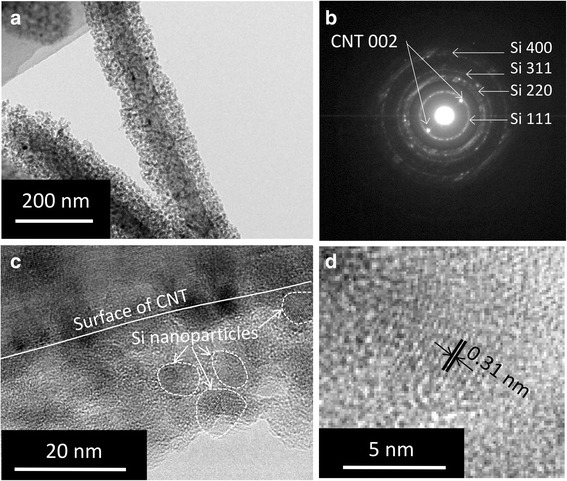
**a** TEM image of Si-coated CNTs. **b** SAED pattern of Si-coated CNTs. **c** Interface between CNT and silicon layer. **d** High magnification image of Si nanoparticles

TEM images of the Si-coated CNTs are presented in Fig. 3. Each CNT was coated with a 20-nm-thick layer (Fig. 3a) that consists of nanoparticles about 5 nm in diameter (Fig. 3c). The SAED pattern (Fig. 3b) from the Si-coated CNTs also indicates that the coated fibers consist of Si and CNTs; no impurities were observed. Figure 3d shows a high-resolution image of nanoparticles. Lattice fringes with 0.31 nm spacing, which corresponds to the (111) planes of crystalline silicon, were observed. Thus, we conclude that the nanoparticles are crystalline Si. This finding regarding the magnesiothermic reduction of silica to produce Si nanoparticles is in good agreement with a previously reported result [27]. In addition, there is good contact between the Si and the CNT at the interface, and no reacted layer was observed.

**Fig. 4:**
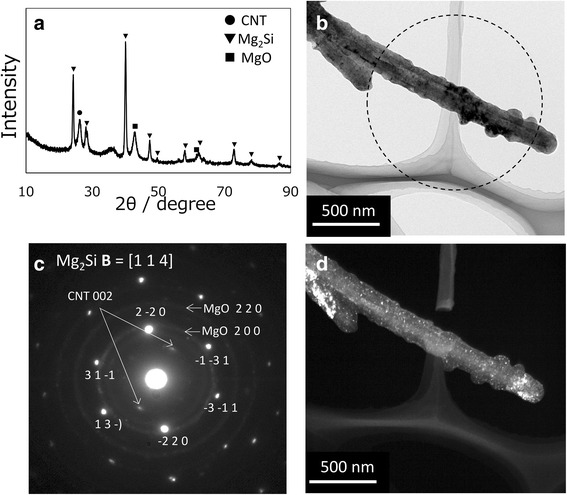
XRD profile and TEM images of the nanofiber after the reaction of Si with magnesium. **a** XRD profile of the nanofiber. **b** Bright field image. **d** The corresponding dark-field image taken with the MgO 200 diffraction spots. The area surrounded by *dashed line* in **b** indicates the observed area of SAED pattern. **c** SAED pattern of the nanofiber

Figure 4 shows an XRD profile of the nanofibers and TEM images of a single nanofiber after the reaction of Si-coated CNTs with magnesium powder. The XRD profile (Fig. 4a) indicates the presence of Mg_2_Si, CNTs, and MgO after the reaction; there was no evidence of unreacted Mg powder in the reacted mixture. Each CNT was coated with a layer about 60 nm thick (Fig. 4b). The SAED pattern in Fig. 4c, taken from the area indicated by the dashed circle in Fig. 4b, exhibits diffraction spots of Mg_2_Si. These results suggest the whole coating consists of an Mg_2_Si single crystal and confirm that Mg_2_Si-coated CNTs were successfully obtained.

Mg_2_Si is synthesized according to the following reaction:

2 $$ 2\mathrm{Mg}\left(\mathrm{l},\mathrm{g}\right)+\mathrm{S}\mathrm{i}\left(\mathrm{s}\right)\to\ {\mathrm{Mg}}_2\mathrm{S}\mathrm{i}\left(\mathrm{s}\right). $$

However, the mechanism of the formation of single crystal Mg_2_Si on CNTs is not well understood. The very slow cooling rate of 1 K/min during the liquid-solid phase reaction [25] may contribute to the growth of the single crystal. In Fig. 4c, in addition to the diffraction spots of Mg_2_Si, there are diffuse diffraction rings from MgO, which means nanocrystalline MgO also exists in the coating. Figure 4d shows a dark-field image of the nanofiber in Fig. 4b taken with the MgO 200 diffraction spots, and nanosized white spots are uniformly dispersed in the image of the matrix. Thus, we conclude that these white spots are MgO nanocrystals.

The possible reason for the presence of MgO within the Mg_2_Si single crystal is discussed as follows. In this study, in order to synthesize Mg_2_Si-coated CNTs, magnesium powder was reacted with Si-coated CNTs at a molar ratio of Si to magnesium of 3:1. In spite of an excess amount of magnesium relative to Si used for the reaction, unreacted magnesium was not observed after the synthesis process. Thus, some unknown reaction may occur during the heating process in addition to the synthesis of Mg_2_Si. One possibility for this reaction involves the presence of a small amount of silica, even after the magnesiothermic reduction, which was probably produced by natural oxidation on the surface of the Si nanoparticles visible in Fig. 3 [28, 29]. During the synthesis process of Mg_2_Si, this small amount of silica on the surface of Si nanoparticles is reduced by magnesiothermic reduction according to reaction (1), resulting in the formation of Si and MgO nanoparticles. This additional Si would also react with magnesium. Therefore, as the Mg_2_Si single crystal grows on the CNT, it incorporates MgO nanoparticles. This may be the reason why nanocrystalline MgO is dispersed inside the Mg_2_Si single crystal.

Regarding the thermoelectric properties of Mg_2_Si-coated CNTs, the electrical conductivity was 0.98 S/m, the Seebeck coefficient was −30 × 10^−6^ V/K, and the calculated power factor was 8.94 × 10^−10^ W/mK^2^ at room temperature. It is clear that Mg_2_Si-coated CNTs are n-type thermoelectric nanofibers, although the measured thermoelectric properties are somewhat lacking in accuracy because the measured specimen was actually an Mg_2_Si-coated CNT/epoxy composite. The measured thermoelectric properties are inferior to those of other CNT-based n-type thermoelectric materials [14, 15] and to those of bulk Mg_2_Si [18, 25]; in particular, electrical conductivity was very low. Compared to the measured electrical conductivity of acid-treated CNT/epoxy composite, which was 599 S/m and comparable with reported value of MWCNT compact [30], the electrical conductivity of Mg_2_Si-coated CNT was still extremely low. We believe this poor electrical conductivity is mainly due to the presence of MgO nanoparticles in the Mg_2_Si coating layer [31]. In addition, the contact resistance between Mg_2_Si coatings may be high because Mg_2_Si is a rigid material and the surface of the Mg_2_Si coating is not flat as shown in Fig. 4b. In order to improve the thermoelectric properties of Mg_2_Si-coated CNTs and to develop an n-type flexible TE film that can be used at relatively high temperature, it is necessary to reduce the amount of MgO nanoparticles in the Mg_2_Si coatings by optimizing the processing parameters. Also the volume and morphology of the Mg_2_Si coating should be controlled, not only to decrease the resistance between nanofibers by reducing the amount of contact between Mg_2_Si coatings and by increasing the amount of Mg_2_Si-CNT and CNT-CNT contact, but also to improve the flexibility of TE films by improving the deformability of the composite nanofibers. A study to address these problems is presently underway.

## Conclusions

We have successfully synthesized Mg_2_Si-coated CNTs through the combined processes of the sol-gel method, magnesiothermic reduction, and liquid-solid phase reaction for use as a flexible TE material at temperatures above 500 K. The synthesized Mg_2_Si coating was an approximately 60-nm-thick single crystal, although it was not single phase as it contained MgO nanoparticles. The synthesized Mg_2_Si-coated CNTs exhibited n-type TE behavior confirming that n-type thermoelectric composite nanofibers were successfully obtained. Future work would involve improving the electrical conductivity and thermoelectric properties by optimizing the process parameters.

## Abbreviations

CNT: Carbon nanotubes
CVD: Chemical vapor deposition
EDX: Energy dispersive X-ray spectroscopy
FE-TEM: Field-emission transmission electron microscopy
Mg_2_Si: Magnesium silicide
MgO: Magnesium oxide
PEDOT:PSS: Poly(3,4-ethylenedioxythiophene):poly(styrenesulfonate)
SAED: Selected-area electron diffraction
Si: Silicon
TE: Thermoelectric
TEOS: Tetraethyl orthosilicate
XRD: X-ray diffraction
